# Dexmedetomidine as an adjuvant to local anesthetics in brachial plexus blocks: A meta-analysis of randomized controlled trials

**DOI:** 10.1097/MD.0000000000005846

**Published:** 2017-01-27

**Authors:** Yongmei Ping, Qigang Ye, Wenwei Wang, Pingke Ye, Zhibin You

**Affiliations:** a Department of Anaesthesiology, Maternal and Children Hospital of Lishui City, Lishui; b Department of Anaesthesiology, Taizhou First People's Hospital and Huangyan Hospital of Wenzhou Medical University, Taizhou, China.

**Keywords:** anesthesia adjuvants, brachial plexus block, dexmedetomidine

## Abstract

**Background:**

Brachial plexus block (BPB) for upper extremity surgery provides superior analgesia, but this advantage is limited by the pharmacological duration of local anesthetics. Dexmedetomidine (DEX) as a local anesthetics adjuvant for BPB has been utilized to prolong the duration of the nerve block in some randomized controlled trials (RCTs) but is far from unanimous in the efficacy and safety of the perineural route. Hence, an updated meta-analysis was conducted to assess the efficacy and safety of DEX as local anesthetic adjuvants on BPB.

**Methods:**

A search in electronic databases was conducted to collect the RCTs that investigated the impact of adding DEX to local anesthetics for BPB. Sensory block duration, motor block duration, onset time of sensory and motor block, time to first analgesic request, the common adverse effects were analyzed.

**Results:**

Eighteen trails (1014 patients) were included with 515 patients receiving perineural DEX. The addition of DEX prolonged the duration of sensory block (WMD 257 minutes, 95%CI 191.79–322.24, *P* < 0.001), motor block (WMD 242 minutes, 95%CI 174.94–309.34, *P* < 0.001), and analgesia (WMD 26 6 minutes, 95%CI 190.75–342.81, *P* < 0.001). Perineural DEX also increased the risk of bradycardia (OR=8.25, 95%CI 3.95–17.24, *P* < 0.001), hypotension (OR = 5.62, 95%CI 1.52–20.79, *P* < 0.01), and somnolence (OR = 19.67, 95%CI 3.94–98.09, *P* < 0.001). There was a lack of evidence that perineural DEX increased the risk of other adverse events.

**Conclusions:**

DEX is a potential anesthetic adjuvant that can facilitate better anesthesia and analgesia when administered in BPB. However, it also increased the risk of bradycardia, hypotension, and somnolence. Further research should focus on the efficacy and safety of the preneural administration of DEX.

## 1 Introduction

Brachial plexus block (BPB), which is a common means of nerve block, has been widely used in hand surgery and orthopedic surgery.^[1]^ With the intervention of ultrasound and nerve stimulator technology, efficacy and safety of the BPB were also greatly improved.^[1]^ BPB provides important advantages compared with general anesthesia, including excellent pain control, reducing side-effects, and attenuating postoperative pain.^[2]^ However, the duration of analgesia has been estimated to be 8 to 14 hours even though long-acting local anesthetics (LAs) are commonly used.^[3]^ Although continuous catheter-based nerve blocks can extend postoperative analgesia, their placement requires additional time, cost, and increases the risk of infection and neurological complications.^[4]^ Recently, several adjuvants to local anesthetics, such as opioids,^[5]^ epinephrine,^[6]^ clonidine,^[7]^ magnesium,^[8]^ midazolam,^[9]^ dexamethasone,^[10]^ and buprenorphine^[11]^have demonstrated to prolong the duration of analgesia of nerve blocks with varying degrees of success.

Dexmedetomidine (DEX) is a highly selective alpha-2 adrenergic receptor agonist.^[12]^ In previous clinical studies, the administration of intravenous DEX has shown to produce significant opioid sparing effects, as well as a decrease in inhalational anesthetic requirement.^[12]^ Recently, DEX as an local anesthetic adjuvants has been the subject of increasing interest as the potential to prolong blockade duration.^[13]^ One recent meta-analysis^[14]^ concluded that DEX as neuraxial adjuvant decreased postoperative pain intensity with 24 hours, prolonged analgesic duration by approximately 7 hours, and accelerated onset of sensory block. Does DEX also facilitate better anesthesia and analgesia on BPB? Many clinical studies had reported that perineural DEX could facilitate a better effect on BPB,^[15,16]^ others could not arrive at the similar conclusions.^[17,18]^ The previous meta-analysis^[19]^ showed peripheral DEX prolong the duration of motor block and the time to first analgesic request. Additionally, the effect on sensory block and the onset of block were not significant. However, the results might be biased. First, the data presented in the meta-analysis combine that obtained after both peripheral nerve and neuraxial block. There are differences in the mechanism of action of local anesthetics using these 2 techniques. Second, the study only extends into the 4 trials (259 patients) which related to BPB, and there were high levels of heterogeneity, which will influence the pooled result to some extent. In order to gain refined evidence, we performed a systematic review and meta-analysis to assess the effect of DEX as local anesthetic adjuvants on BPB.

## 2 Materials and methods

### 2.1 Literature search

In accordance with Quality of Reporting of Meta-analyses (QUORUM) guidelines and the recommendations from Cochrane Collaboration, a systematic search strategy was performed on MEDLINE, EMBASE, Cochrane Central Register of Controlled Trials, EBSCO and Cochrane Library using brachial plexus, peripheral nerve block, regional anesthesia, axillary, interscalene, supraclavicular, infraclavicular, nerve block, dexmedetomidine, medetomidine, and Precedex as key words. Retrieval time was from the time of database establishment to June 2016. The reference lists of included articles were also reviewed. This is a meta analysis. Thus, ethical approval was not necessary and the informed consent was not given.

### 2.2 Eligibility criteria

Studies were included if they met the following criteria: (1) RCTs; (2) comparison between LAs with perineural DEX and LAs without DEX in single-injection BPB regional anesthesia for forearm and hand surgery; (3) adult patients without general anesthesia; and (4) in English.

Studies were excluded if they were (1) non-RCTs; (2) continuous or repeated nerve blocks; (3) DEX administered through nonperineural route or without LAs.

### 2.3 Data extraction and outcome assessment

All articles were reviewed by 2 reviewers (QY and YP) to independently evaluate the methodological quality of the included RCTs using the Cochrane Collaboration's risk of bias assessment tool. When they disagreed with each other, disagreements were either discussed to reach a consensus between the 2 reviewers or decided by 2 other reviewers (WW and KY). The extracted information included main author, publication year, surgical location, sample size, BPB types, nerve localization techniques, perineural DEX dosage (shown as dosages per average body weight or per 70 kg ),^[7]^ LA dosage, and types. The data was shown as median and interquartile range (IQR) with estimated mean and standard deviation (SD) in accordance with Cochrane Handbook (IQR ≈ 1.35 SD).^[20]^ The sensory block duration was defined as primary outcomes. The secondary outcome included duration of motor block, onset time of sensory and motor block, time to first request of analgesic, and DEX-related adverse effects.

### 2.4 Statistical analysis

Meta-analysis was performed using STATA 12.0 (Stata Corp) and RevMan 5.2 (Cochrane Collaboration). Data used weighted mean differences (WMD) and odds ratios (ORs), presented as 95% confidence intervals (CI). The χ^2^ test was used for heterogeneity analysis, and heterogeneity was assessed by *I*^2^. If *I*^2^ < 50%, the fixed effects model was used; if *I*^2^ > 50%, the heterogeneity was assessed. Subgroup analyses were used to assess the impact of BPB types, nerve localization technique, DEX dosage and LA dosage, and types on BPB. Multivariate regression was used to assess the difference of routes, dosage, and localization technique on BPB. Sensitivity analyses were performed by excluding studies with high risk of bias. If the heterogeneity cannot be identified, data were pooled for the random-effects model. *P* < 0.05 was considered as statistically significant. Begg's funnel plot and Egger's test were used to determine potential publication bias.

## 3 Results

### 3.1 Search results

**Figure 1:**
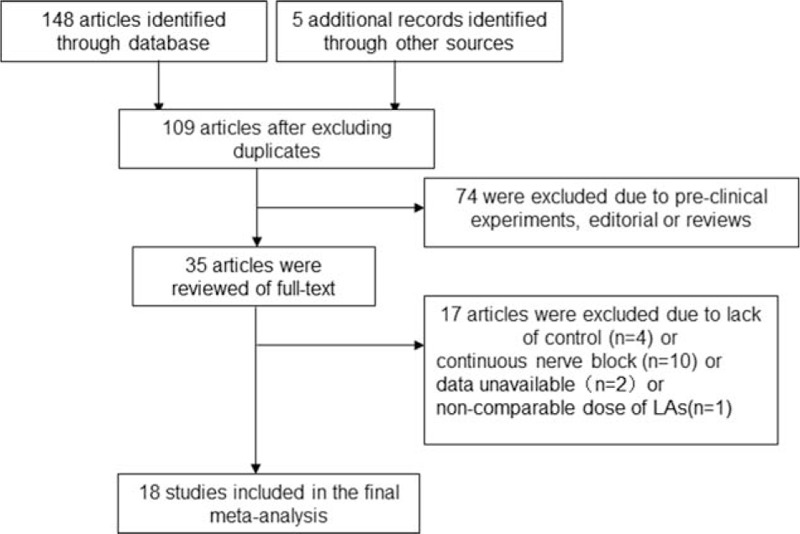
Diagram of the review process.

The search process was shown in Fig. 1. There were 153 articles identified in the search process. After screening of titles and abstracts and review of full text, 118 articles were excluded because they were duplicate publications, preclinical experiments, editorials, or reviews. Upon further review, 16 articles were excluded due to lack of controls, continuous nerve block, or unavailable data. One article was excluded due to noncomparable LA dosages.^[21]^ Finally, 18 studies were included.^[16,22–38]^

### 3.2 Characteristics of included studies

Detailed characteristics of included studies were shown in Table 1. Of the 18 studies, 10 studies were from India (52.6%), 3 studies were from South Korea (15.8%), and 5 studies were from other countries (26.3%). There were 6 studies of DEX administered through the axillary route, 9 studies through the supraclavicilar route, and 3 studies through the infraclavicular route. And, 12 studies used a nerve stimulator for nerve localization and 6 used ultrasound. DEX was used in combination with long-acting LAs (bupivacaine in 3 studies, levobupivicaine in 3 studies, and ropivacaine in 10 studies) or intermediate-acting LAs (mepivacaine in 1 study and lidocaine in 1 study). The DEX dosage varied from 50 to 100 μg, with fixed dosage in 12 studies and varied dosage in 6 studies. Seventeen trials tested 1 dose of DEX and 1 trail tested 2 doses.

**Table 1:**
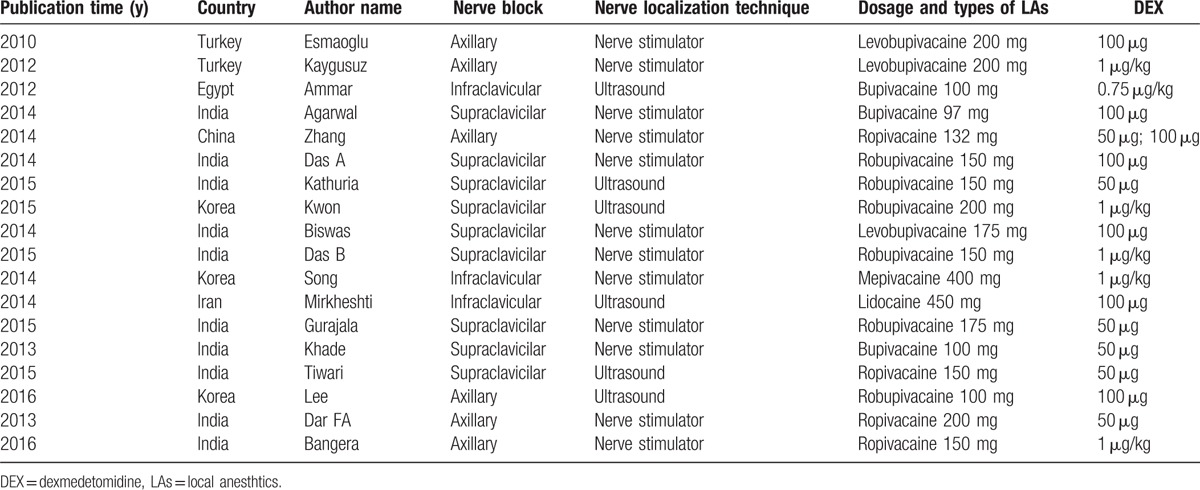
General characteristics of included studies.

### 3.3 Assessment of quality and bias

**Figure 2:**
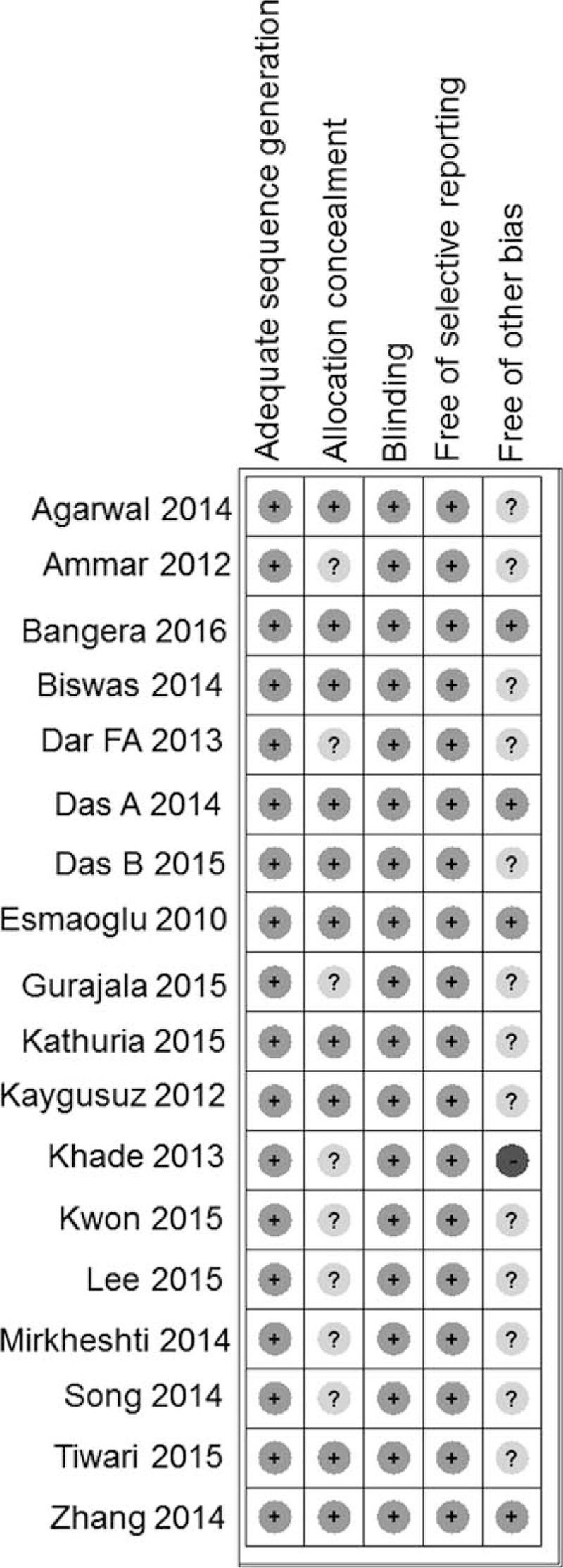
Methodological quality and bias risk. Green circle = low bias risk, red circle = high bias risk, yellow circle = unclear bias risk.

To determine the study quality and potential bias, risk of assessment was performed. There were 17 studies that the reported randomization procedure in detail and 10 studies that mentioned the allocation concealment. All trails reported their double-blinded administration. However, there was 1 study^[34]^ of high bias risk due to ambiguous definition of onset and duration of sensory and motor block. The risk of bias was detailed in Fig. 2.

### 3.4 Duration of sensory block

**Figure 3:**
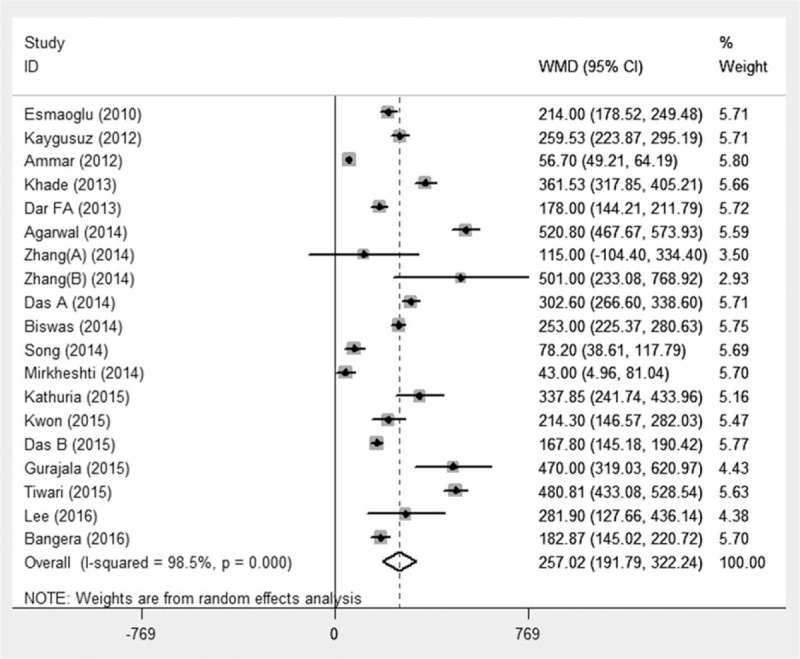
Duration of sensory block. Pooled analysis showed significantly prolonged duration of sensory block in the DEX group compared with those without DEX (WMD 257 min, 95%CI 191.79–322.24, *P* < 0.001). CI = confidence interval, DEX = dexmedetomidine, WMD = weighted mean difference.

There were 515 patients in the DEX group and 499 patients in the control group among all 18 studies.^[16,22–38]^ The definition of duration of sensory block varied from time interval between LA administration or complete sensory block to complete sensory recovery. Pooled analysis showed significantly prolonged sensory block duration when combined with DEX (WMD 257 minutes, 95%CI 191.79–322.24, *P* < 0.001), compared with that without DEX, as shown in Fig. 3). However, the heterogeneity was significant among pooled studies (*I*^2^ = 98.5%). When subgrouped by DEX dosage (50 μg or > 50 μg), it showed no effects of different nerve localization technique on different routes of BPB. There was no heterogeneity (*I*^2^ = 0%) for the infraclavicular route; however, there was significant heterogeneity for the axillary route (*I*^2^ = 68.4%) and the supraclavicilar route (*I*^2^ = 95.9%). When subgrouped by LA types, it showed that the estimated prolongation of sensory block was 282.65 minutes with long-acting LAs and 60.16 minutes with intermediate-acting LAs. Sensitivity analysis was performed by excluding studies of high bias risk. Nonetheless, all the meta-analyses results were not affected by studies with high risk bias. Multivariate regression was performed to identify the potential source of heterogeneity. The result showed that DEX dosage, LAs, administration route, or localization techniques all did not contribute to the heterogeneity, as shown in Table 2. The above indicated that DEX as local anesthetic adjuvants on BPB significantly prolonged the duration of sensory block.

**Table 2:**
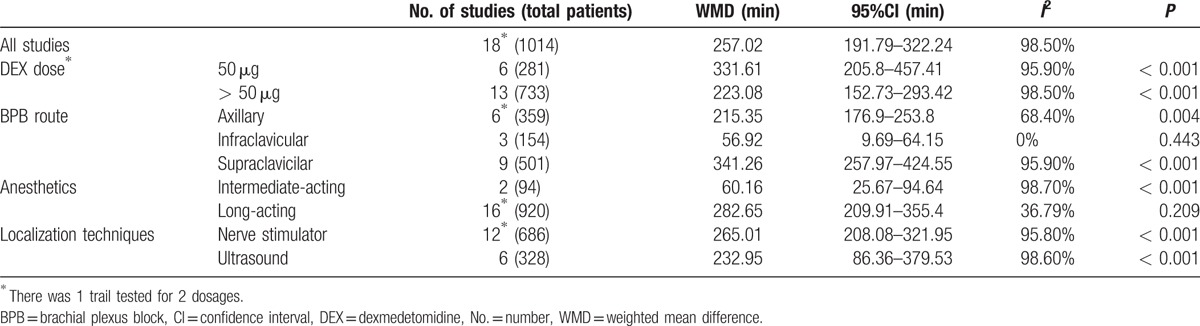
Subgroup analyses of DEX on duration of sensory block.

### 3.5 Duration of motor block

**Figure 4:**
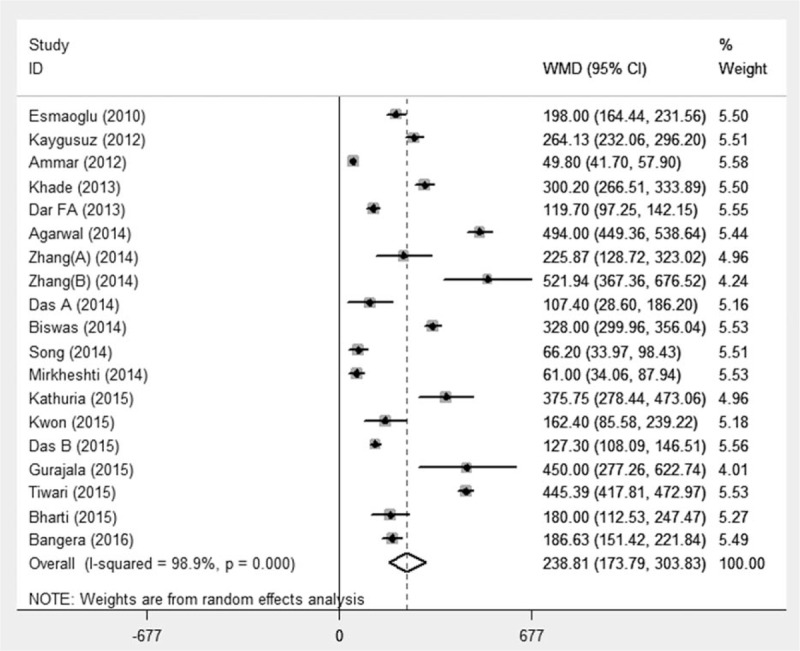
Duration of motor block. Pooled analysis showed significantly prolonged duration of motor block in the DEX group compared with those without DEX (WMD 238 min, 95%CI 173.79–303.83, *P* < 0.001). CI = confidence interval, DEX = dexmedetomidine, WMD = weighted mean difference.

There were 498 patients in the DEX group and 482 patients in the control group among 17 studies,^[16,22–35,37,38]^ whereas 1 study was excluded without detailing motor block. The definition of duration of motor block varied, mostly defined as the time interval between LA administration to complete motor sensory recovery. Pooled analysis showed the overall estimated motor block duration of DEX as adjuvants in BPB was 238 minutes (95%CI 173.79–303.83, *P* < 0.001) with significant heterogeneity (*I*^2^ = 98.9%), as shown in Fig. 4. Further subgroup analyses of LA types, DEX dosage, administration route, and localization techniques, as well as sensitivity analyses did not contribute to the heterogeneity. The above indicated that DEX as local anesthetic adjuvants on BPB significantly prolonged the duration of motor block.

### 3.6 Time to nerve block onset

**Figure 5:**
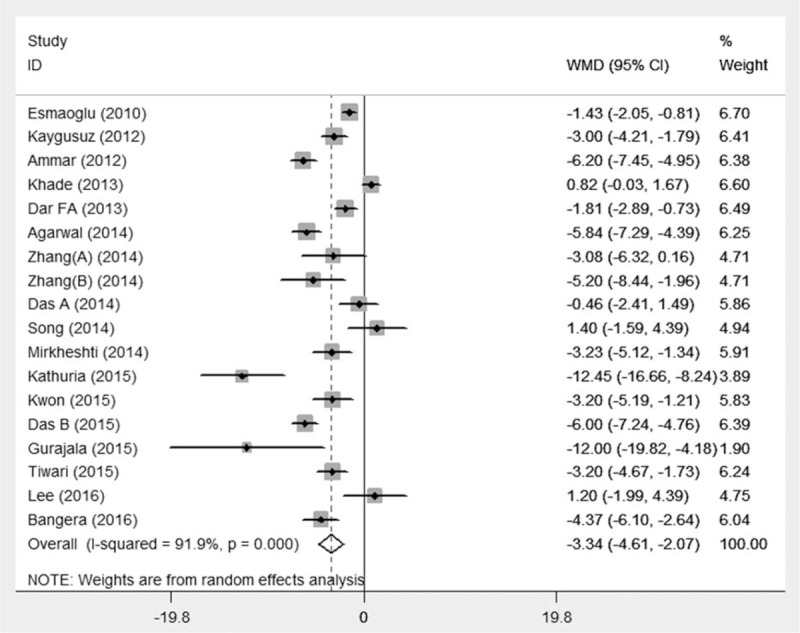
Time to onset of sensory and motor block. Pooled analysis showed perineural DEX could accelerate the onset of sensory block (WMD –3.34 min, 95%CI –4.61 to –2.07, *P* < 0.001). CI = confidence interval, DEX = dexmedetomidine, WMD = weighted mean difference.

**Figure 6:**
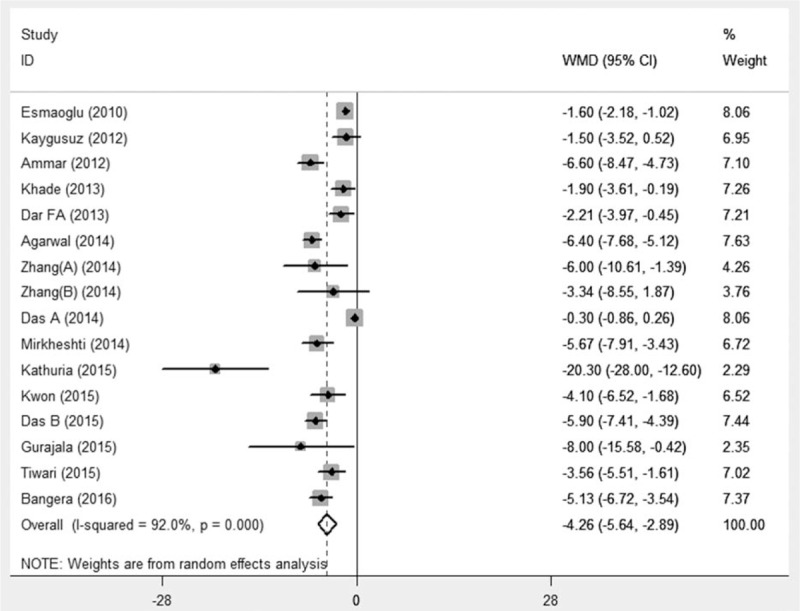
Time to onset of motor block. Pooled analysis showed perineural DEX could accelerate the onset of motor block (WMD –4.26 min, 95%CI –5.64 to –2.89, *P* < 0.001). CI = confidence interval, DEX = dexmedetomidine, WMD = weighted mean difference.

The onset of sensory and motor block was reported in 18^[16,22–38]^ and 15^[16,22–28,30,32–35,37,38]^ studies, respectively. Pooled analysis showed that perineural DEX accelerated the onset of sensory block (WMD –3.34 minutes, 95%CI –4.61 to –2.07, *P* < 0.001) (Fig. 5) and onset of motor block (WMD –4.26 minutes, 95%CI –5.64 to –2.89, *P* < 0.001) (Fig. 6) with significant heterogeneity (*I*^2^ = 91.9% and *I*^2^ = 92%, respectively). Further, subgroup analyses showed that BPB types, nerve localization techniques, DEX dosages, and LA types did not influence the pooled results with high heterogeneity. The above indicated that DEX as local anesthetic adjuvants on BPB significantly accelerated the time to onset of sensory and motor.

### 3.7 Duration of analgesia

**Figure 7:**
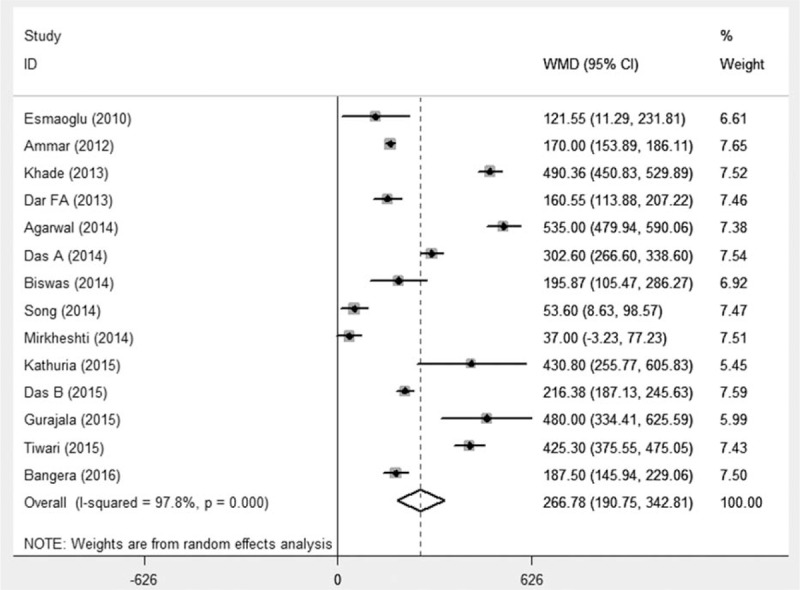
Duration of analgesia. Pooled analysis showed DEX as adjuvant could prolong the duration of analgesia (WMD 266 min, 95%CI 190.75–342.81, *P* < 0.001). CI = confidence interval, DEX = dexmedetomidine, WMD = weighted mean difference.

There were 14 studies^[16,23,24,26,27,29–37]^ that evaluated the duration of analgesia. Totally 815 patients were involved, with 408 of them received perineural DEX. The duration of analgesia was defined as time interval from LAs administration or successful sensory block to additional analgesics. The pooled analysis showed that DEX as adjuvants significantly prolonged duration of analgesia (WMD 266 minutes, 95%CI 190.75–342.81, *P* < 0.001) with significant heterogeneity (*I*^2^ = 97.8%), as in Fig. 7. Further subgroup analyses showed that LA types, DEX dosage, administration route, and nerve localization techniques would not change the overall results.

### 3.8 The incidence of adverse events

The incidence of bradycardia and hypotension were, respectively, described in 11^[16,24–26,28,30,31,33–37]^ and 10^[16,22,25–28,31,33–36]^ studies. Pooled analysis showed that perineural DEX increased the risk of bradycardia (OR = 8.25, 95%CI 3.95–17.24, *P* < 0.001) and hypotension (OR = 5.62, 95%CI 1.52–20.79, *P* < 0.01). In 4 studies,^[23,24,34,37]^ perineural DEX increased the risk of somnolence (OR = 19.67, 95%CI 3.94–98.09, *P* < 0.001). Pooled analysis showed no difference between DEX and the controlled group in terms of other adverse events, such as nausea, vomiting, or hypoxemia. All of these analyses showed no heterogeneity (*I*^2^ = 0); thus, the fixed-effects model was used, as in Table 3.

**Table 3:**
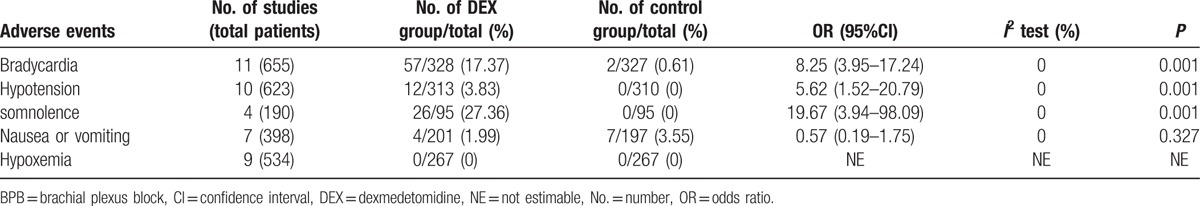
The incidences of adverse events.

### 3.9 Publication bias

**Figure 8:**
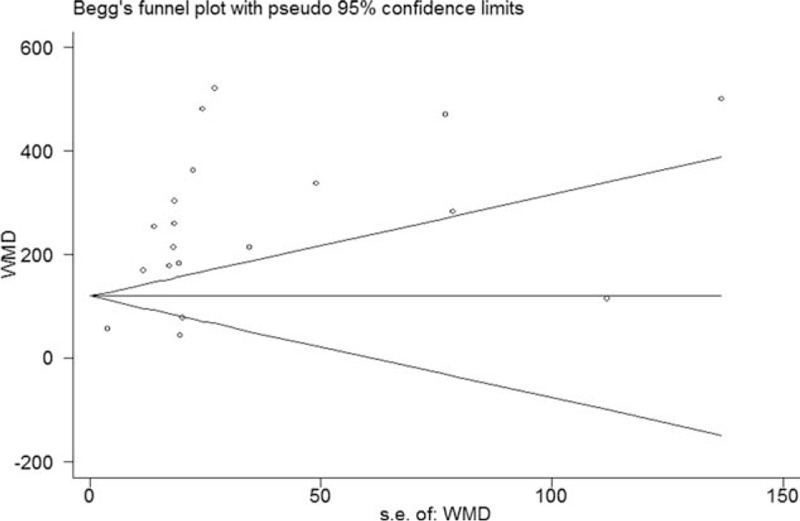
Begg's funnel plot and Begg's test. It showed significant publication bias in the primary outcome (duration of sensory block) (*P* = 0.006). WMD = weighted mean difference.

To determine publication bias, Begg's test and Egger's test were used. Both Begg's test (*P* = 0.006) and Egger's test (*P* = 0.001) indicated potential publication bias in the primary outcome (duration of sensory block), as in Fig. 8.

## 4 Discussion

Our systematic review and meta-analysis indicated that DEX as local anesthetic adjuvants on BPB significantly prolonged the duration of sensory, motor block, and analgesia, as well as accelerated the time to onset of sensory and motor. However, these were at a cost of an increased risk of bradycardia, hypotension, and somnolence.

DEX, a highly selective alpha-2 adrenergic receptor agonist, is of strong sedative, analgesic, anesthetic sparing effects when used in general anesthesia.^[12]^ In the meantime, it can also be used as a perineural adjuvant to facilitate better anesthesia and analgesia.^[12,39]^ The hypothesized mechanisms of DEX administration in the peripheral nerve block are as follows: DEX inhibits the function of sodium channels and neuronal potassium current^[40–42]^ and blocks the hyerpolarization-activated cyclic nucleotide-gated channels, resulting in the enhancement of activity-dependent hyperpolarization^[43]^ and leading to the inhibition of substance *P* release in the nociceptive pathway at the dorsal root neuron.^[44]^

Our study included 18 studies on the efficacy and safety of DEX as local anesthetic adjuvants for BPB with high quality control. However, pooled analyses showed high heterogeneity. Further subgroup analyses, sensitivity analyses, and multivariate regression were performed to identify the origin of heterogeneity; however, they failed to change the heterogeneity.

Pooled analyses showed that DEX's duration of sensory block was longer in combination with long-acting LAs than that with intermediate-acting LAs (282 vs 60 minutes). However, it might be biased due to that only 2 studies included intermediate-acting LAs. One animal study^[45,46]^ and 1 clinical study^[25,47]^ indicated that the efficacy of perineural DEX administration in combination with LAs was in a dosage-dependent manner. However, the dosage-dependent manner cannot be identified, potentially due to the high heterogeneity.

Several limitations of our study could potentially contribute to the observed heterogeneity. First, our study might be influenced by publication bias based on Bebb's funnel plot and Egger's test. Although the facilitatory effects of perineural DEX on BPB may be encouraging, only the intravenous route for DEX is approved by the U.S Food and Drug Administration (FDA) and Health Canada. Most of included trials were performed in developing countries and published in nonanesthesia journals. Second, varied BPB types, nerve localization techniques, DEX dosages, and LA types, as well as varied definitions of onset and duration of sensory and motor block, and unclear adverse events all could contribute to the methodological heterogeneity.

The safety of the preneural administration of DEX is currently the research hotspot. Pooled analyses showed that DEX would increase the incidence of bradycardia and hypotension. It might be resulted from the inhibition of DEX on sympathetic outflow and release of norepinephrine through alpha-2 subtype receptors.^[12,39]^ However, the bradycardia and hypotension were transient and could be reversed by atropine or ephedrine. Our study also showed that perineural DEX would increase the occurrence of somnolence, which is likely due to systemic reabsorption. Recently, Fritsch et al^[3]^ showed that the highest plasma DEX level was 0.64 ng/mL with 150 μg DEX and ropivacaine at 30 minutes after block, and the plasma DEX level was 0.29 ng/mL at 180 minutes. Potts et al^[48]^ showed the effective DEX plasma level ranged from 0.4 to 0.8 ng/mL. Therefore, the DEX plasma levels were relatively low and were decreasing over time.

Neurological deficit was not observed in our study. Brummett et al^[46]^ showed the neurotoxicity of DEX when the dosage of perineural DEX up to 40 μg/kg in rat and it might even attenuate the acute perineural inflammation induced by bupivacaine. Erdivanli et al^[49]^ also echoed the above results and showed no pathohistological changes of nerve system after the intrathecal administration. Fritsch et al^[3]^ showed no neurological sequelae after administration of DEX and ropivacaine for BPB at postoperative 7 day and 28 day. Though the safety of preneural administration of DEX is encouraging, information concerning delayed neurological effects is still lacking.

In conclusion, this meta-analysis showed that DEX, as local anesthetic adjuvants for BPB, would significantly prolong the duration of sensory block, motor block and analgesia, and accelerate the time to onset of sensory and motor block with increased risk of bradycardia, hypotension, and somnolence. Further research should focus on the long-term safety of the DEX preneural administration.
